# Supplementary figures and images for: MYH11 rare variant augments aortic growth and induces cardiac hypertrophy and heart failure with pressure overload
Source: PLoS Genet. 2025 Jul 14;21(7):e1011394. doi: 10.1371/journal.pgen.1011394 (PMC12273954; doi:10.1371/journal.pgen.1011394)

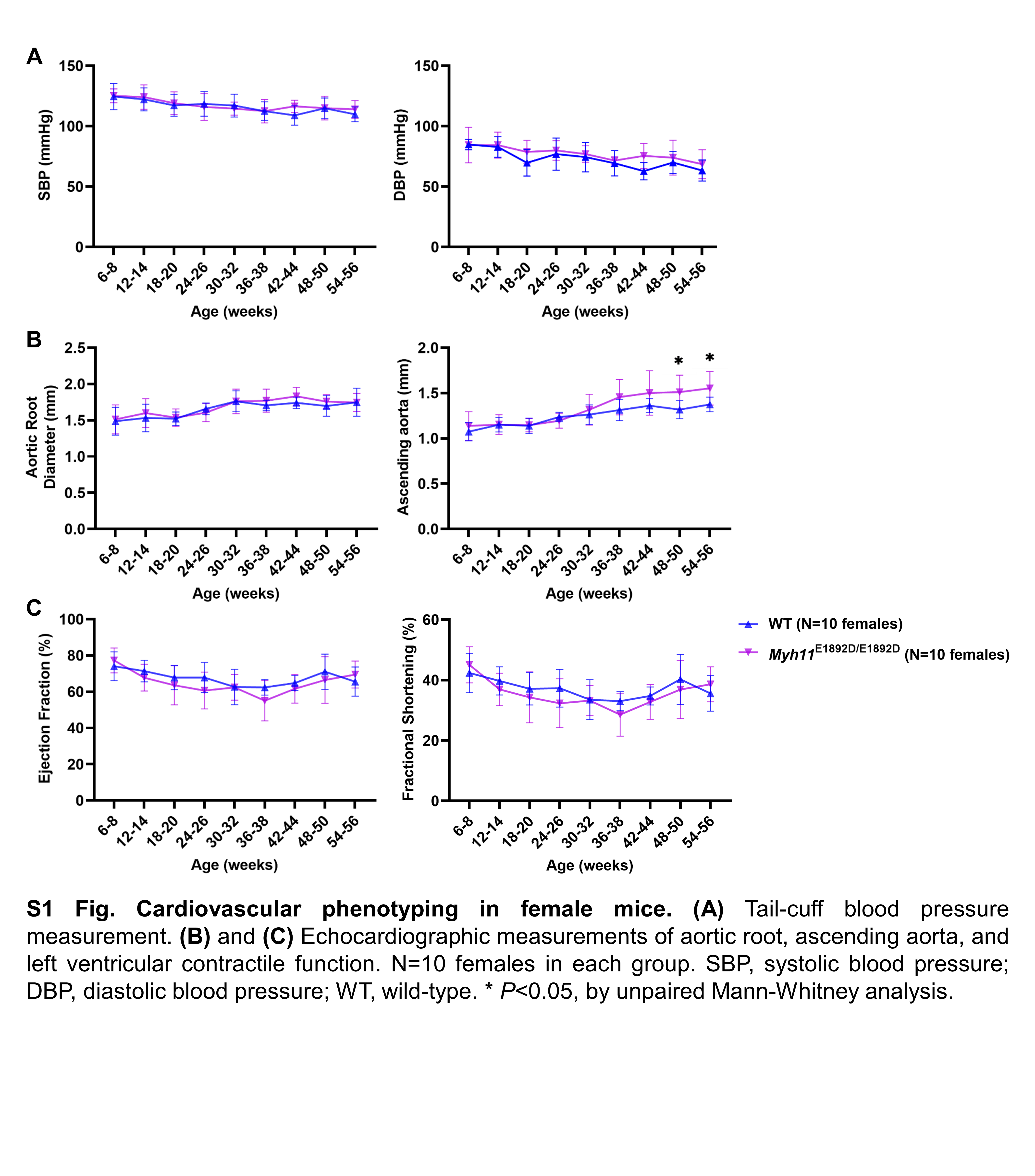

Supplement: S1 Fig — (A) Tail-cuff blood pressure measurement. (B) and (C) Echocardiographic measurements of aortic root, ascending aorta, and left ventricular contractile function. N = 10 females in each group. SBP, systolic blood pressure; DBP, diastolic blood pressure; WT, wild-type. * P < 0.05, by unpaired Mann-Whitney analysis. (TIF) [file pgen.1011394.s001.TIF]

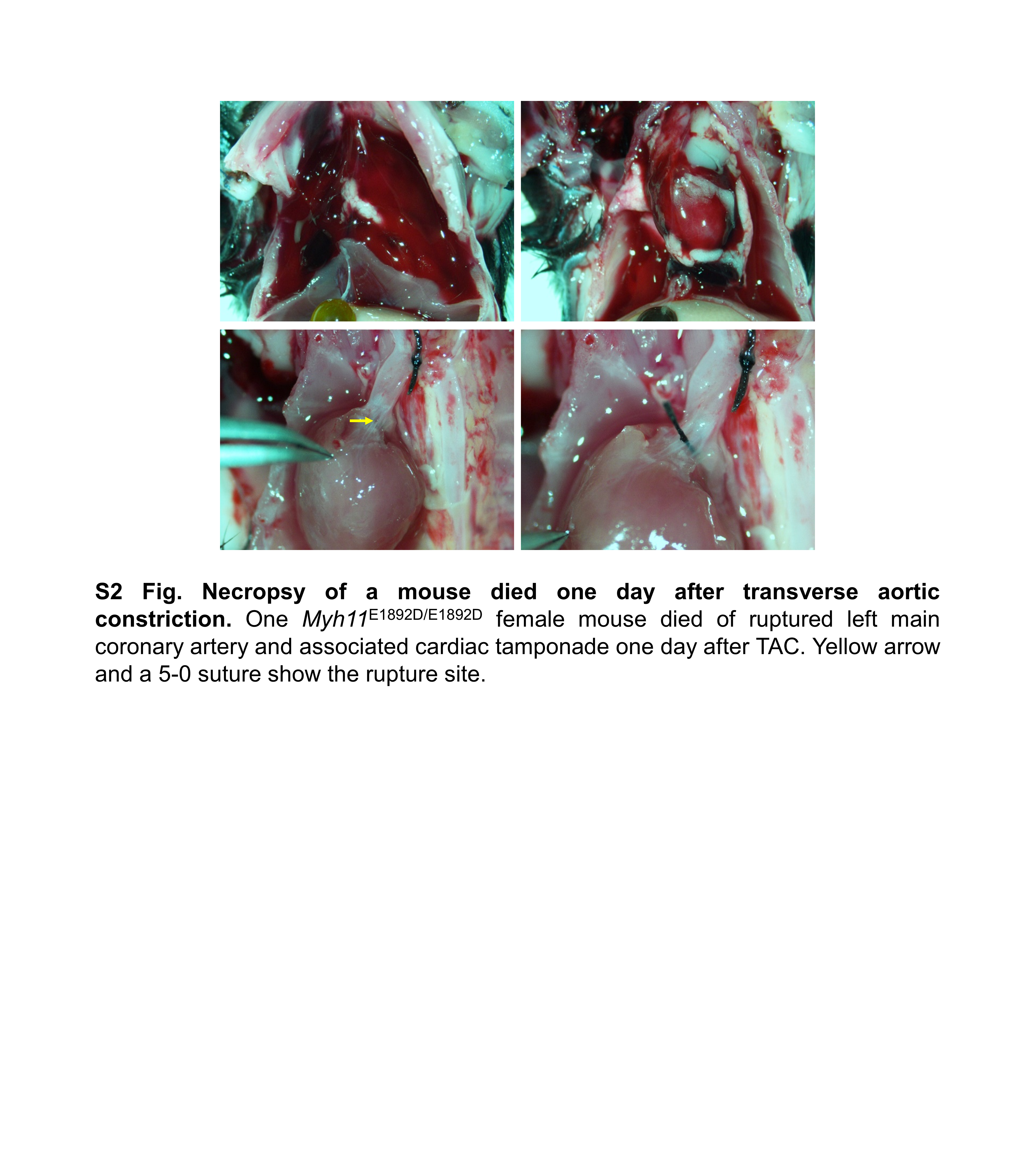

Supplement: S2 Fig — One Myh11E1892D/E1892D female mouse died of ruptured left main coronary artery and associated cardiac tamponade one day after TAC. Yellow arrow and a 5–0 suture show the rupture site. (TIF) [file pgen.1011394.s002.TIF]

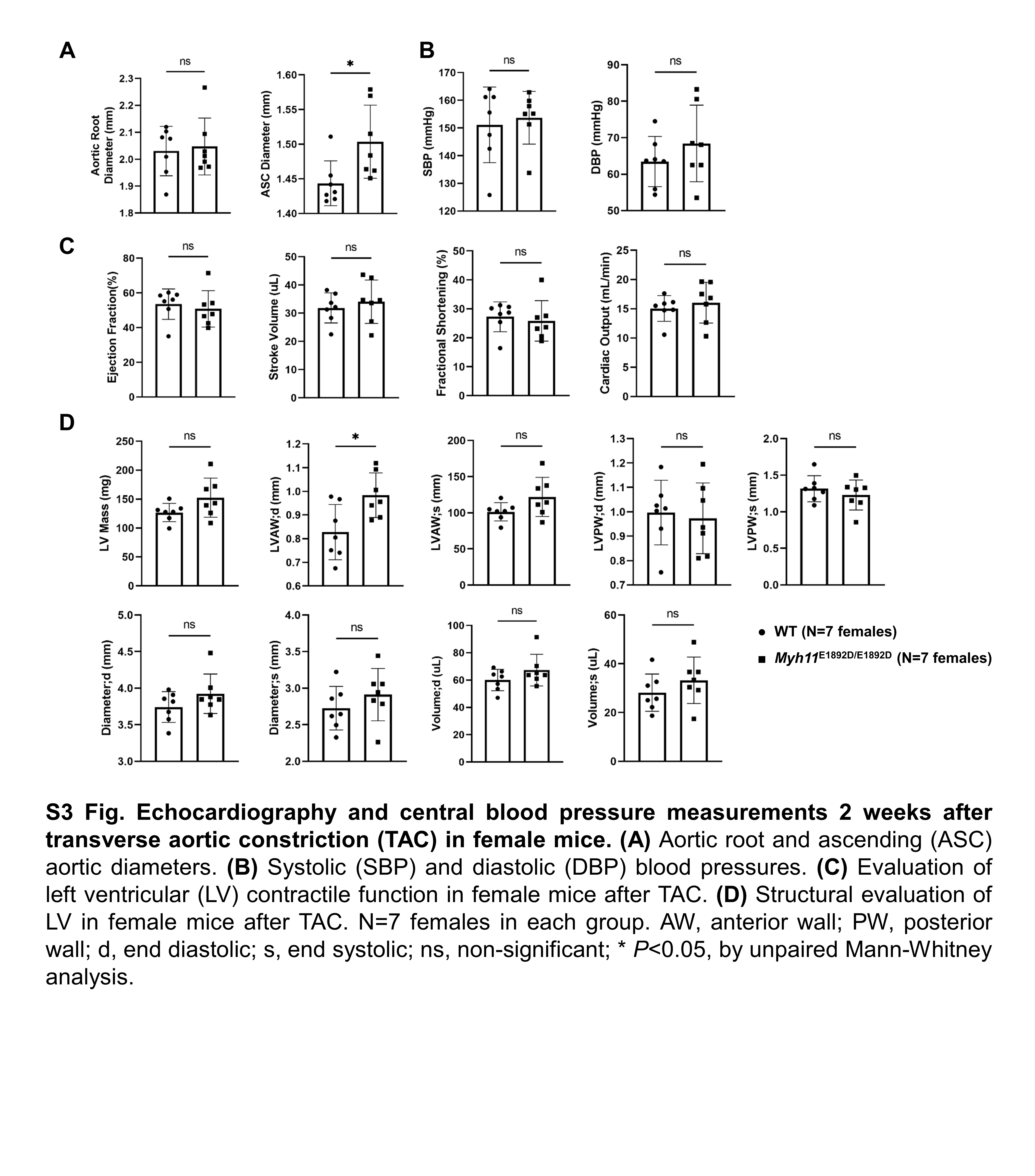

Supplement: S3 Fig — (A) Aortic root and ascending (ASC) aortic diameters. (B) Systolic (SBP) and diastolic (DBP) blood pressures. (C) Evaluation of left ventricular (LV) contractile function in female mice after TAC. (D) Structural evaluation of LV in female mice after TAC. N = 7 females in each group. AW, anterior wall; PW, posterior wall; d, end diastolic; s, end systolic; ns, non-significant; * P < 0.05, by unpaired Mann-Whitney analysis. (TIF) [file pgen.1011394.s003.TIF]

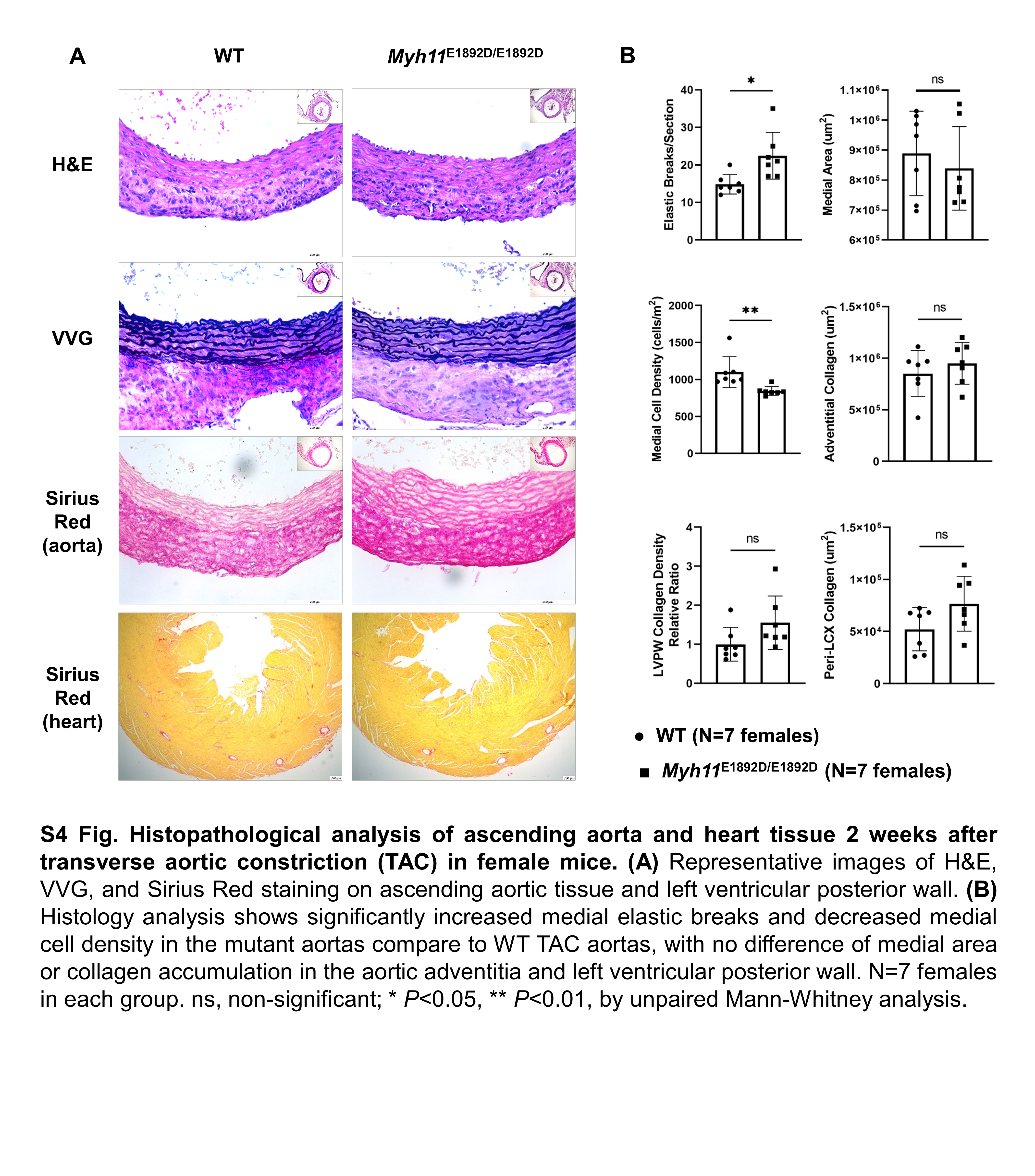

Supplement: S4 Fig — (A) Representative images of H&E, VVG, and Sirius Red staining on ascending aortic tissue and left ventricular posterior wall. (B) Histology analysis shows significantly increased medial elastic breaks and decreased medial cell density in the mutant aortas compare to WT TAC aortas, with no difference of medial area or collagen accumulation in the aortic adventitia and left ventricular posterior wall. N = 7 females in each group. ns, non-significant; * P < 0.05, ** P < 0.01, by unpaired Mann-Whitney analysis. (TIF) [file pgen.1011394.s004.TIF]

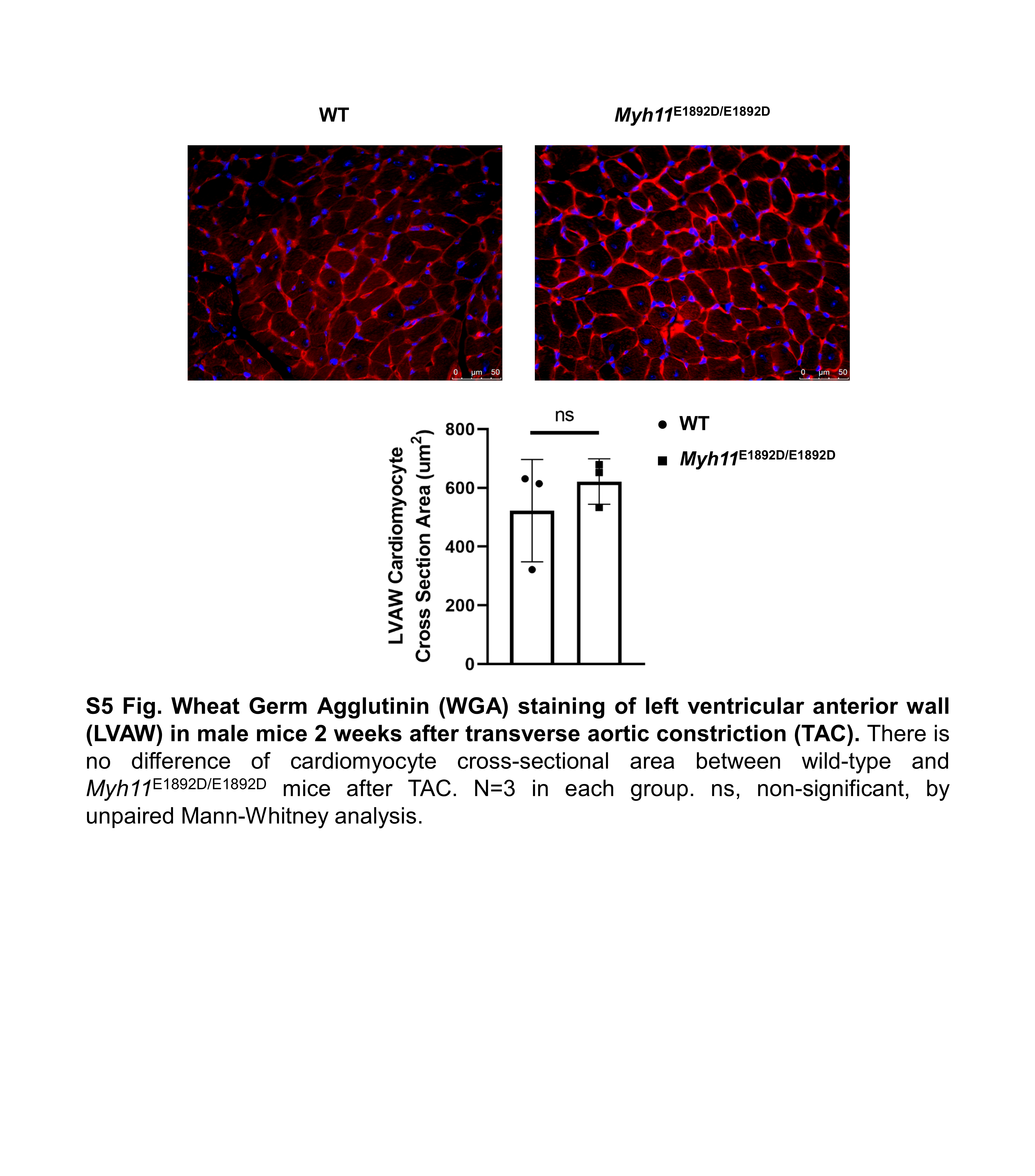

Supplement: S5 Fig — There is no difference of cardiomyocyte cross-sectional area between wild-type and Myh11E1892D/E1892D mice after TAC. N = 3 in each group. ns, non-significant, by unpaired Mann-Whitney analysis. (TIF) [file pgen.1011394.s005.TIF]

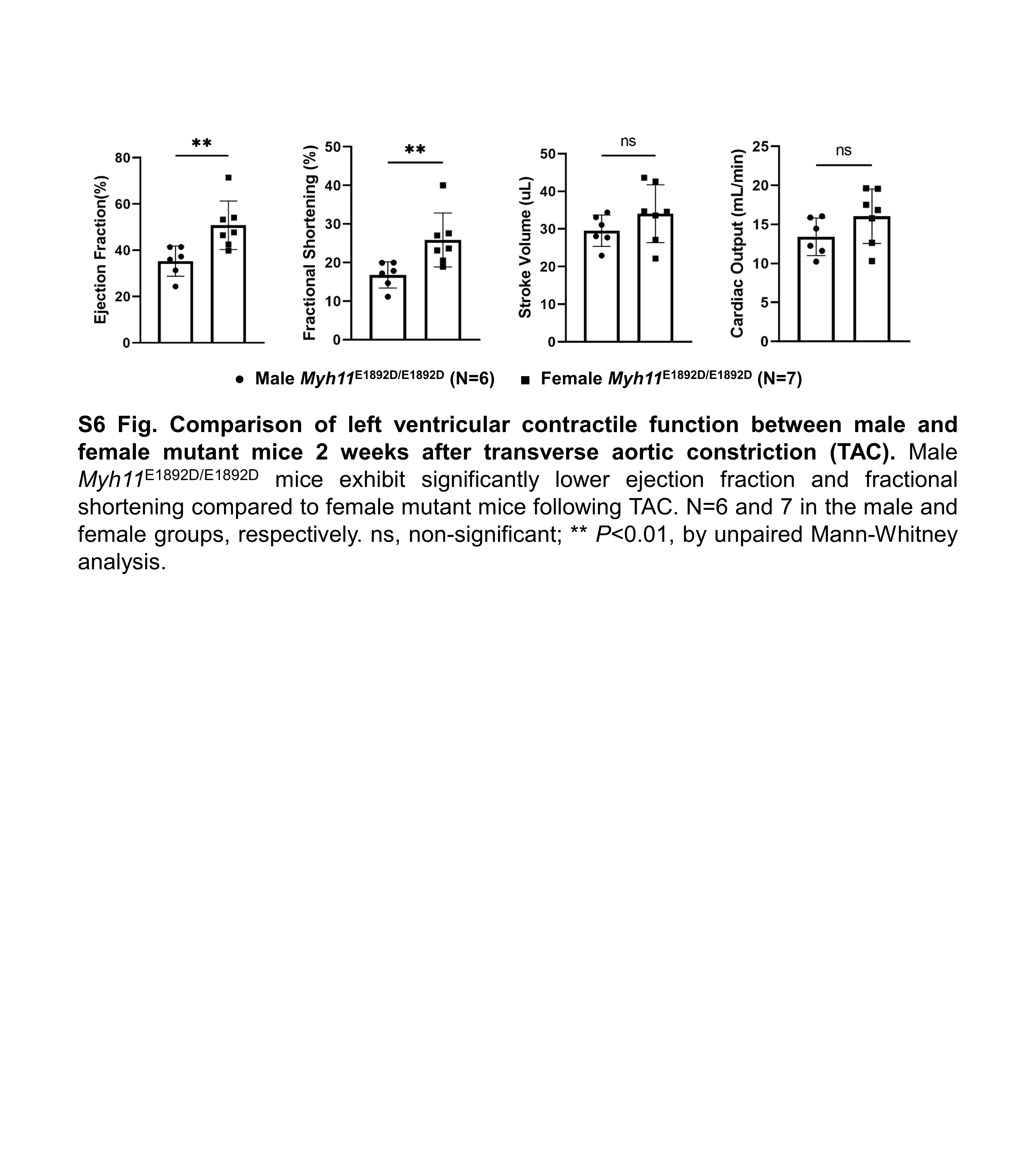

Supplement: S6 Fig — Male Myh11E1892D/E1892D mice exhibit significantly lower ejection fraction and fractional shortening compared to female mutant mice following TAC. N = 6 and 7 in the male and female groups, respectively. ns, non-significant; ** P < 0.01, by unpaired Mann-Whitney analysis. (TIF) [file pgen.1011394.s006.TIF]
