# Supplementary material for: MYH11 rare variant augments aortic growth and induces cardiac hypertrophy and heart failure with pressure overload
Source: PLoS Genet. 2025 Jul 14;21(7):e1011394. doi: 10.1371/journal.pgen.1011394 (PMC12273954; doi:10.1371/journal.pgen.1011394)
Supplement: Attachment — Submitted filename: Response to the Reviewers.docx [file pgen.1011394.s011.docx]

**Reviewer #1:**

*Zhou et al investigated the effects of the MYH11 p.Glu1892Asp variant, a rare variant of uncertain significance, on cardiovascular phenotypes in mice. The authors used genomic editing to create mice with this specific variant (Myh11E1892D/E1892D) and subjected them to transverse aortic constriction (TAC) to simulate pressure overload. While these mutant mice exhibited normal cardiovascular parameters under baseline conditions, TAC induced significant aortic enlargement, elastic fragmentation, and cardiac hypertrophy, particularly in male mice. These male mutant mice also showed impaired left ventricular function, reduced ejection fraction, stroke volume, and increased left ventricular mass due to posterior wall thickening. The findings suggest that the MYH11 variant contributes to adverse cardiovascular remodeling and heart failure under pressure overload conditions, highlighting the importance of smooth muscle cells in these processes.
This manuscript presents a well-structured and thorough investigation into the effects of a rare MYH11 variant on cardiovascular remodeling under pressure overload conditions. The study is commendable for its use of advanced genomic editing techniques to create a relevant mouse model, providing valuable insights into the functional consequences of the MYH11 p.Glu1892Asp variant. The experimental design is robust, with comprehensive cardiovascular phenotyping and detailed histological analyses demonstrating the variant's impact on aortic enlargement and cardiac hypertrophy. The authors also highlight important sex-specific differences in the response to pressure overload, adding a novel dimension to the understanding of MYH11-related pathologies.
1. The only thing missing from the manuscript is the authors need to indicate if the rare variant found in the proband is homozygous or heterozygous. Otherwise, this reviewer believes that the manuscript should be accepted with this minor modification.*

**We thank the reviewer for their positive comments on our manuscript and now clearly state in the manuscript that the *MYH11* variant is heterozygous in the proband.**

**Reviewer #2:**

*In this manuscript, the authors investigate the impact of a rare variant (p.Glu1892Asp) in the MYH11 gene identified in a patient with an aortic root dilatation using a knock-in mouse model. Through genomic editing, the authors generated Myh11E1892D/E1892D mice and performed cardiovascular assessments on mutant and wild-type (WT) controls. The results indicate that mutant mice of both sexes were largely comparable to WT mice in terms of growth, blood pressure, and heart function up to 13 months of age, with the exception of significant enlargement of the ascending aorta in older female Myh11E1892D/E1892D mice compared to female WT controls. The mice were then subjected to transverse aortic constriction (TAC) to induce hypertension. While TAC increased blood pressure in both groups, mutant mice showed a greater degree of ascending aortic enlargement and elastic fiber fragmentation. In male Myh11E1892D/E1892D mice, there was a notable reduction in ejection fraction, stroke volume, fractional shortening, and cardiac output compared to male WT mice two weeks after TAC. Additionally, left ventricular mass significantly increased, primarily due to posterior wall thickening, with cardiomyocyte hypertrophy and elevated collagen deposition in the myocardium and surrounding arteries. The authors conclude that these findings highlight the sex-specific clinical variability associated with the rare MYH11 variant. Overall, the study presents useful insights for the field.*

**Thank you for the positive comments on our manuscript.**

*While the rationale for most methodologies is well-explained, several aspects require clarification and improvements.*

*1. Major comments:*

*1) The results on pages 5-6 focus on the proband with the identified variant. However, this section lacks supporting data. Including some data on aortic root dilation or variant sequencing results would strengthen the findings beyond the text descriptions.*

**The proband requested that no additional clinical details be provided in the manuscript.**

*2) The identified VUS is common (0.6%) in the European population, making it relatively unlikely that it is a disease-causing VUS. Since the proband has pectus excavatum, could the aortic root dilation be associated with this condition rather than the VUS MYH11 p.Glu1893Asp variant? Is there any available information regarding potential cardiovascular phenotypes associated with this variant in the European population?*

**DNA from the proband underwent genome sequencing and only the VUS in *MYH11* was identified. As mentioned in the Discussion, *MYH11* is a loci in genome wide association studies for coronary artery disease and arrythmias, but not cardiac dysfunction or thoracic aortic disease.**

*3) Figure 1: The figure legend title states that it is a homozygous mutation in the mice (Myh11E1892D/E1892D). But the sequencing in A on the right side clearly shows that it is heterozygous with the presence of a G and a C peak. This is confusing requiring clarification. The authors should include sequence data of both the heterozygous and the homozygous mice because they used both in the paper.*

**Thank you for pointing out this error. We have updated the representative DNA sequencing results in Figure 1A to include sequencing of wild-type, heterozygous, and homozygous mice, and illustrates both the mutation and synonymous change that was introduced to limit editing.**

*4) The cardiovascular phenotype of 13-month-old male and female mice was analyzed in Figure 1. The authors observed significant enlargement of the ascending aorta in female homozygous mutant mice compared to wild-type females, but this phenotype was not present in males. However, for further analyses in Figure 2A involving smooth muscle cell (SMC) contractility in aortae using myograph analysis, the authors utilized 3 male and only 1 female mice at 10 months of age. It is unclear why the authors combined sexes for this analysis, especially given the aortic dilation observed in females but not males in Figure 1 and then later the severe phenotype in male mice after TAC in Figure 4. This is especially confusing considering the authors provide a detailed discussion on sex-specific cardiovascular phenotypes (Pages 10-11). Moreover, the sex of the wt, het and homo mice used in Figure 2B at 6-month age is not specified. The rationale for changing the analysis time point to 10 and 6 months as opposed to the initial time point of 13 months is not explained.*

**We agree with the reviewer and Figure 1A now only includes male mice. Since the revisions of this manuscript needed be returned within 6 months and these studies were done by our collaborators, we did not have time to study female mice separately. In Figure 2B, both males and females were included in the immunoblot assay on ascending aortic tissues and there were no alterations in contractile protein levels between the wildtype and mutant. The sex of the mouse for each sample is indicated in the figure.**

*5) Figures 3 and 4: It is not clear from the figure legend whether the histological data provided in 3A, 3C, and 4E is for male or female mice or both combined. If it is only for male mice, then the female mice data should be provided. If both sexes were combined, then given the sex-specific differences observed in Figures 1 and 4, analyses of the two sexes should be conducted separately for wild-type and Myh11E1892D/E1892D mice in 3A, 3C and 4E. The sample size for male and female mice in these figures is not indicated and should be provided.*

**Since heart remodeling and failure were observed only in male mutant mice, the cardiovascular phenotyping data + TAC in Figures 3 and 4, is only male mice as indicated in the text and figure legends. Data on TAC-induced cardiovascular remodeling in females is in Supplementary Figure 3. We have incorporated pathological data from heart and aortic tissues of female mice in Supplementary Figure 4. We have also clarified the sex and sample size in each figure.**

*6) Throughout the manuscript, it is unclear what statistical tests were used for each analysis. This is particularly important in cases where the sample size is n=2. The statistical methods should be clearly stated in the figure legends, and a minimum sample size of n=3 is necessary to ensure reliable conclusions. The sample size should be increased in experiments where n=2 or less for each sex per group.*

**To address this comment, we have added the statistical tests used for each analyses in the figure legends. Regarding sample sizes, we included ascending aortic tissues from mice of both sexes and increased the sample size to ≥5 animals per group for the immunoblot assay in Figure 2B.**

*7) The description of collagen quantification and elastic fiber fragmentation based on picrosirius-stained images is missing under the methods section and should be included.*

**Thank you for pointing out this omission. We have now included a detailed description of these analyses under the Histopathology subsection in the Materials and Methods section.**

**Reviewer #3:**

*In the enclosed manuscript, Zhou et al. have undertaken the important task of studying one of a growing number of genetic VUS identified in a typical clinical encounter with a patient with aortopathy. With the increasing use of genetic testing in patients with aortic aneurysm/dissection, these variants create clinical challenges for affected patients and family members.*

*Here, the authors investigate the effects of MYH11 p.Glu1892Asp variant on aortic function in a murine model. Interestingly, mice homozygous for the E1892D variant showed normal aortic diameters, protein expression of typical contractile markers, and response to vasoactive substances compared to wild-type controls. There was, in other words, no overt aortic phenotype in Myh11^E1892D/E1892D mice. Nevertheless, in a provoked central hypertension model with transverse aortic constriction, these mice showed a greater degree of aortic remodeling, elastin degradation, and aortic root diameter. Taken together, this reviewer interprets the E1892D variant as insufficient to promote aortopathy in isolation, but as a disease accelerant in the setting of a second insult (e.g., hypertension). The authors also report functional changes in left ventricular function (reduced), hypertrophy (increased), and enriched perivascular fibrosis in E1892D mice.*

*I commend the authors for a thorough investigation of the effect of this clinically identified VUS on aortic function in a mouse model. I have the following comments/questions.*

*1. Contractile function appears to be preserved in E1892D mice at baseline, however the histologic changes in TAC treated male mice appears to suggest a heightened "synthetic" phenotype in response to provocation. There are multiple reports of aortic smooth muscle cell phenotype change in aneurysm and hypertension/TAC models. Can the authors assess (either with histologic or PCR methodology) WT and E1892D aortas at baseline and with TAC to assess for recognized markers of SMC phenotype shift to determine if the E1892D variant somehow "primes" cells for progression to a deranged identity in response to stimulus.*

**The fact that contractile protein levels do not change in the mutant compared to wildtype mice indicates that the smooth muscle cells do not de-differentiate, suggesting that these cells are not switching to a “synthetic” phenotype. To further address this comment, additional histological analyses of the ascending aortas following TAC were done. Our findings revealed a significant decrease in medial cell density in mutant aortas of both sexes (shown in Fig. 3 and S4, respectively). Together with the increased medial area, these results suggest that the mutant smooth muscle cells do hypertrophy in respond to TAC to a greater degree that the wildtype smooth muscle cells.**

*2. Like the authors, I find the changes in left ventricular function between E1892D mice and WT in response to TAC striking, however I find the mechanistic explanation for this finding and its relevance to human disease difficult to follow. Is this finding attributed to some change in aortic stiffness/resistance (beyond BP measurements), coronary vascular function (as evidence by remodeling in these vessels), or some other mechanism? Multiple plausible explanations are presented in the discussion, however none of these mechanisms are tested in the treated tissues.*

**We agree that there are multiple plausible explanations, some of which are reviewed in the Discussion, and we are in the process of exploring possible mechanisms for the left ventricular functional changes. We can only conclude that the mutant smooth muscle cells are responsible for the left ventricular changes but given the number of possible causes, these investigations are beyond the scope of this manuscript.**

*3. Changes in aortic histology and diameter following TAC did not translate to aortic deaths in E1892D mice; do the authors propose that this is the result of short follow up time/constraints of the TAC model (which also necessarily produces a ventricular phenotype) or a suggestion of a fairly mild aortic phenotype in homozgous E1892D animals.*

**We hypothesize that this is a limitation of the TAC model, since TAC also induces cardiac failure over time and this failure is augmented in the male mutant mice. At the same time, the female mutant did not show increased heart failure when compared to female wild-type mice and the female mutant mice did not dissection.**

**Reviewer #4:**

*In this manuscript, the authors describe the cardiovascular phenotype of a novel genetically modified mouse strain that models a variant of unknown significance in the MYH11 gene. While at baseline there are no significant differences between mutant and control mice (except for a mild ascending aortic dilation in aged female mutant mice), adding additional stress by TAC surgery did provoke a stronger phenotype in the MYH11 mutant mice. The strength of this study is that a new mouse model was generated to test the in vivo effects of the MYH11 variant on cardiovascular function, before and after surgically induced hemodynamic stress. Several issues remain to be addressed in order to support the relevance of the conclusions made by the authors.*

*1. It is not clear whether the proband was heterozygous for the MYH11 p.Glu1892Asp VUS, or whether it was present on both alleles. In the former case, how do the authors reconcile this with the fact that only the homozygous mutant mice appear to show a phenotype (based on the data in Fig. 2)? Were any aortic or cardiac measurements performed in heterozygous mice at baseline and/or after TAC?*

**The *MYH11* p.Glu1892Asp** **variant identified in the proband is heterozygous, and we have updated this information in the manuscript.**

**For almost all genetic mouse models of thoracic aortic disease, the pathogenic variants identified in humans need to be homozygous in the mice to cause thoracic aortic disease (PMID: 35614093 - *Myh11*^K1256del^; 10097121 - *Fbn1*^mgR/mgR^). Exceptions are *Fbn1* ^C1041G/+^ (PMID: 16601194) and *Prkg1*^R177Q/+^ (PMID: 31387997); mice in the heterozygous state have slowly growing thoracic aortic aneurysms that do not progress to acute aortic dissections. Therefore, given the mild phenotype in the *Myh11*^E1892D/E1892D^ mice, we do not expect a phenotype in the heterozygous mice. We have completed analyses of 5 wild-type and 2 *Myh11*^E1892D/+^ male mice with TAC, and there is no aortic or cardiac phenotype in these mice. Unfortunately, we did not have time before the deadline for the resubmission of these revisions to complete these additional studies.**

*2. In addition, the E1892D variant is currently classified as benign or likely benign in Clinvar (as acknowledged by the authors), based on multiple submission highlighting that it is present in 0.5% of chromosomes in gnomAD and ExAC, represents a conservative amino acid change, and is predicted to be benign based on multiple in silico models. How would the authors propose to re-classify the variant based on the current data? Should it be considered as a variant leading to predisposition for aortic disease and/or cardiomyopathy in combination with other risk factors?*

**As mentioned, our data indicate that this variant may be a low penetrant variant for thoracic aortic disease, and possibly only contribute to disease in the presence of hypertension. Therefore, the variant should be classified as a “risk” variant and not a pathogenic variant (PMID: 29961567). The protocols to classify rare variants as low penetrant, risk variants are being defined by ClinGen.**

*3. Which mechanism do the authors propose leads from the conservative substitution of Glu to Asp at position 1892 in MYH11 to the observed predisposition to aortic dilation and cardiac remodeling after pressure overload? Are there any in vitro studies that support an effect of this modification on protein function? The (sparse) data in Fig.2 suggests that the mutation does not appear to have an effect on protein expression – although this would need to be confirmed with more samples (see other comment below).*

**We do not know if this variant disrupts protein function, although the studies in Figure 2A indicates that it does not significantly disrupt force generation by smooth muscle cells. It may disrupt polymerization or interactions with other proteins or turnover of the heavy chain of the contractile unit, or many other potential effects that we have not assessed.**

*4. The studies referred to in line 66-67 made use of a fragment of the MYH11 promoter to drive Cre expression, which indeed was restricted to SMC. However, can the authors exclude that the endogenous MYH11 promoter, which might be affected by additional long-range enhancers or other genomic modulators, does not lead to leaky expression in other cell types at any point during development? This is important to check as one of the main hypotheses of the manuscript is that defects in vascular SMCs in the heart can contribute to pressure overload-induced cardiac hypertrophy and failure.*

**We would like to clarify that the mouse model used in our study carries the point mutation introduced via CRISPR/Cas9 gene editing, rather than the Cre/LoxP system. Therefore, concerns regarding potential Cre-mediated recombination leakage do not apply in this case.**

*5. The manuscript lacks any information on how the Myh11 p.E1892D mouse line has been generated. Fig. 1 panel A seems to contain some markings relevant to the CRISPR strategy used, but they are not explained in the manuscript. Was the synonymous missense variant c.5679C>A introduced during CRISPR genome editing? Did the authors check for off-target effects of CRISPR/Cas editing?*

**The c.5679C>A synonymous variant was engineered as a silent mutation to prevent re-cutting of the CRISPR reagents during mutagenesis and was not an off-target mutation. To confirm the integrity of the modifications, we performed DNA sequencing on both the initial founder animal and the first-generation offspring derived from crossing the founder with the wild-type C57BL/6J strain. It was determined that the c.5679C>A variant was present in the founder and co-segregated with the intended c.5676G>C mutation to the next generation.**

*6. Based on the role of MYH11 variants in cardiac rhythm (mentioned in the discussion, lines 268-271): did the authors perform any ECG measurements in the E1892D mutant mice? Was basal heart rate (as measured during echocardiography, but unfortunately not shown in the manuscript) different from wild-types?*

**We did not observe any differences in basal heart rate or any evidence of cardiac arrhythmia during echocardiographic assessments in the mutant mice of both sexes. We have added a sentence to the Results to indicate there was no evidence of disrupted cardiac rhythm.**

*7. The western blot data shown in Fig. 2 panel B only includes 2 samples for both the WT and mutant groups. This is not sufficient to be able to draw any meaningful conclusions, and needs to be repeated with larger numbers.*

**To address this concern, we have increased the sample size to ≥5 per group for the immunoblot assay in Figure 2B using ascending aortic tissues from mice of both sexes.**

*8. Finally, and importantly, a large part of the discussion focuses on many potential pathways that may be affected in the MYH11 mutant animals (IGF-1, PI3K/Akt/mTORC1, apoptosis, renin-angiotensin-aldosterone system, TGF-beta, inflammation, …). However, this is all very speculative and not based on any actual data collected in the current study. While it cannot be expected that the authors explore all pathways in detail in this study, the manuscript would strongly benefit from additional molecular data which might implicate any of these pathways in the observed phenotype. This could be achieved either by additional western blot data (including from animals that underwent the TAC procedure), QPCR, immunostaining, or potentially more unbiased methods including (single cell/nucleus) RNA-sequencing.*

**We acknowledge the importance of exploring the mechanistic pathways involved. At the same time, our discussion illustrates that there are many possible causes that may contribute to the observed phenotype and it may take many years to identify the correct cause for the left ventricular failure.**

*9. Minor comments:*

*1) The rationale to test TAC in this model instead of Angiotensin II and/or L-NAME infusion, as was done in other models investigating MYH11 variant function, is not clear.*

**We chose the TAC model over angiotensin II infusion or L-NAME plus a high-salt diet because TAC offers a rapid and reproducible method for inducing pressure overload that does not involve treating the mice with drugs. The drugs have the potential to contribute to the aortic and cardiac remodeling, thus complicating what exactly is driven by increased biomechanical force. Given our focus on assessing the cardiovascular phenotype due to a genetic variant, TAC was the “cleanest” system to assess the response of the mutant mice to increased biomechanical forces on the cardiovascular system.**

*2) The section on lines 205-210 on FBN1 variants does not seem to fit with the rest of the discussion, it should be made more clear what the point is the authors want to make here.*

**Since *MYH11* is only expressed in SMCs, we can conclude that the cardiac phenotype is driven by SMCs changes due to the *MYH11* variants. In contrast, *FBN1* is expressed in many cells, including cardiomyocytes, therefore, the cardiac dysfunction in Marfan patients may result from the *FBN1* pathogenic variant disrupting cardiomyocyte function directly rather than affecting mutant SMCs. We have revised this section to clarify this point.**

*3) Fig. S2 lacks the top of panels A and B*

**The error that occurred during the PPT-to-PDF conversion has been corrected.**
