# Supplementary material for: MYH11 rare variant augments aortic growth and induces cardiac hypertrophy and heart failure with pressure overload
Source: PLoS Genet. 2025 Jul 14;21(7):e1011394. doi: 10.1371/journal.pgen.1011394 (PMC12273954; doi:10.1371/journal.pgen.1011394)
Supplement: Attachment — Submitted filename: Rebuttal Letter.docx [file pgen.1011394.s012.docx]

**Reviewer #4:**

*The authors have addressed most of my prior comments satisfactorily, but only two more points remain.*

*1. The most important point relates to my previous question #4, which I’m afraid the authors may have misunderstood. What I meant to say was that in the manuscript the authors use evidence from prior studies which make use of a fragment of the mouse Myh11 promoter to drive Cre expression as a basis to assume that the expression of Myh11 is restricted to SMC. Nevertheless, in the current manuscript the authors indeed performed CRISPR manipulation of the endogenous Myh11 gene. The expression of this gene is however driven by the full-length endogenous promoter (which could be influenced by distal elements and genome topology), and not only by the fragment used to demonstrate SMC-specific expression. My question therefore is whether there is conclusive evidence that the endogenous, genomic Myh11 promoter leads to the exact same expression pattern as the shorter fragment used to drive Cre expression in the prior studies cited by the authors. This is important as a key element of the conclusions put forth in this manuscript is that the observed phenotypes are exclusively SMC-dependent.*

**The *Myh11* cre mice were created to facilitate research in SMCs because of the already established specificity of the *Myh11* expression in SMCs. More recently, single-cell transcriptomic datasets from various tissues consistently show that endogenous *Myh11* expression is specific to SMCs, including early developmental stage (PMID 36575170; 35830816). In response to this comment, we have revised the Introduction (page 4, paragraph 1) to incorporate this additional supporting evidence.**

*2. Thank you for clarifying that the proband is heterozygous for the MYH11 variant. It could be interesting to add a short line in the discussion on the point of using homozygous mutant mice for this study. The authors can indeed highlight that in a number of other mouse models of thoracic aortic disease a relevant phenotype can only be observed when the pathogenic variant or other mutation is biallelic. This does however raise some questions regarding the potential translation from mouse to human and vice versa: does the mouse model have more genetic redundancy? Or is the mouse aorta more resistant to heterozygous genetic defects?*

**We have now included a brief rationale for the use of homozygous mutant mice in the first paragraph of the Discussion. This common requirement for biallelic mutations in murine models likely reflects species-specific differences. While the precise reasons remain incompletely understood, it is possible that the mouse aorta is inherently more resistant to structural compromise from single-allele mutations.**
